# Cervical length following cerclage as a predictor of spontaneous preterm birth

**DOI:** 10.1007/s00404-026-08453-9

**Published:** 2026-05-08

**Authors:** Maya Frank Wolf, Liron Kinog, Ruba Tuma, Lior Lowenstein, Marwan Odeh, Inshirah Sgayer

**Affiliations:** 1 Department of Obstetrics and Gynecology, Galilee Medical Center, Nahariya, Israel; 2 Azrieli Faculty of Medicine, Bar Ilan University, Safed, Israel; 3 Raya Strauss Wing of Obstetrics and Gynecology, Galilee Medical Center, 2210001 Nahariya, Israel

**Keywords:** Transvaginal cervical length, Spontaneous preterm birth, Cervical insufficiency, McDonald cerclage, Prediction

## Abstract

**Objective:**

We aimed to evaluate the association between serial transvaginal cervical length measurements following McDonald cerclage and spontaneous preterm birth (PTB).

**Methods:**

This retrospective study included singleton pregnancies with cerclage performed during 2010–2024. Cerclage was placed prophylactically (*n* = 109) based on obstetric history, or emergently (*n* = 46) due to ultrasound findings. Cervical length was measured by transvaginal ultrasound before and after cerclage, and at 2-week intervals until 32 weeks.

**Results:**

For the prophylactic group, the median cervical length was shorter among those who delivered PTB < 37 weeks (*n* = 23) than term: at 21–22 + 6 weeks (2.5 vs. 3.9 cm, *p* = 0.042), 23–24 + 6 weeks (2.0 vs. 3.4 cm, *p* = 0.016), 25–26 + 6 weeks (3.0 vs. 3.8 cm, *p* = 0.042), and 31–32 + 6 weeks (2.4 vs. 3.4 cm, *p* = 0.015). In multivariable analysis adjusted for history of PTB, progesterone use, and gestational age at cerclage placement, shorter cervical length, at 23–24 + 6 weeks (adjusted odds ratio [aOR] 4.13, 95% confidence interval [CI] 1.23–13.89, *p* = 0.021) and at 25–26 + 6 weeks (aOR 3.39, 95% CI 1.08–10.64, *p* = 0.037), was independently associated with PTB < 37 weeks. Cervical length at 25–26 + 6 weeks was associated with PTB < 32 weeks (aOR 4.76, 95% CI 1.20–19.60, *p* = 0.027). For the emergency group, the median cervical length was shorter among those who delivered < 32 weeks than later, at 23–24 + 6 weeks (1.4 vs. 3.2 cm, *p* = 0.049) and 25–26 weeks (1.5 vs. 2.3 cm, *p* = 0.041).

**Conclusion:**

Serial cervical length monitoring after cerclage provides a clinically relevant prediction of spontaneous PTB.

## What does this study add to the clinical work?

**Table:** 

Serial transvaginal cervical length measurements after cerclage placement were significantly associated with spontaneous preterm birth. Shorter cervical length at 23–24+6 and 25–26+6 weeks independently were independently associated with preterm birth in prophylactic and emergency cerclage groups. This study highlights the prognostic value of cervical surveillance following cerclage in identifying women at continued risk for early delivery.	

## Introduction

Preterm birth (PTB) remains a significant cause of perinatal morbidity and mortality, often resulting from multifactorial conditions including infection, inflammation, placental pathology, and cervical insufficiency [1]. Cervical insufficiency, characterized by painless cervical dilation in the absence of uterine contractions, affects about 1% of pregnancies and is a recognized contributor to second-trimester loss and early PTB [2].

Cervical cerclage is a surgical intervention intended to reduce the risk of PTB in women with structural or functional cervical weakness. Indications for cerclage placement include: a history of mid-trimester pregnancy losses or spontaneous PTB (history-indicated), painless cervical dilation observed during physical examination (exam-indicated), and significant cervical shortening on ultrasound, particularly in women with prior PTB or cervical procedures (ultrasound-indicated) [3].

Measuring cervical length by transvaginal ultrasound (TVS) is an established method for assessing the risk of spontaneous PTB. Cervical shortening has been suggested to reflect ascending infection or inflammation, potentially preceding clinical signs of labor or membrane rupture. [4] However, the placement of a cerclage may alter the cervix anatomically or artificially lengthen it, potentially confounding the predictive value of cervical length measurements post-procedure [5, 6].

Whether cervical length measured after cerclage placement remains a reliable marker for PTB risk is not fully established [6, 7]. The role of routine post-cerclage monitoring of cervical length remains controversial, as evident by the diversity in clinical practices and the limited consensus regarding its predictive value. While some clinicians incorporate serial measurements into surveillance protocols, others question its utility due to inconsistent associations with outcomes [3, 8]. Clarifying its prognostic relevance could support more individualized management, including decisions about hospitalization and the timing of interventions such as corticosteroids or magnesium sulfate. Therefore, this study aimed to evaluate whether routine post-cerclage cervical length monitoring provides a clinically meaningful prediction of spontaneous PTB.

## Methods

### Study design and definitions

This retrospective study included women who underwent cervical cerclage at our tertiary medical center during 2010–2024. Inclusion criteria were singleton pregnancies with McDonald cerclage placement, based on obstetric history or ultrasound findings, and with available serial TVS cervical length measurements. Cerclage indication was categorized according to clinical criteria. Prophylactic cerclage was defined as placement in women with a history of second-trimester pregnancy loss or spontaneous PTB due to painless cervical dilation. Emergency cerclage was defined as placement due to significant cervical shortening (< 25 mm on TVS) in the absence of contractions or ruptured membranes. Women who underwent emergency cerclage were identified by their having progressive shortening during cervical length follow-up, or by their presenting with symptoms such as pelvic pressure, increased discharge, or spotting. Women were also identified when a short cervix was detected incidentally during the routine fetal anatomy scan, which is performed according to the national guidelines. According to our policy, women with a history of spontaneous preterm also underwent serial transvaginal cervical length measurements every 2 weeks, beginning at 16 weeks of gestation, and were offered vaginal progesterone prophylaxis. When cervical shortening progressed despite progesterone treatment, cerclage placement was considered. Exclusion criteria included multiple gestations, major fetal anomalies, physical examination-indicated cerclage, and missing delivery data.

### Collected data

The maternal variables accessed included age, gravidity, parity, smoking status, prior PTB, and pregnancy complications such as gestational diabetes and hypertensive disorders. Procedure-related data included gestational age at cerclage placement, an indication for cerclage (prophylactic [history-indicated] or emergency [ultrasound-indicated]; examination-indicated cerclage was excluded due to very small numbers [*n* = 2]), and serial cervical length measurements. In our center, qualified physicians measure cervical length via TVS, within 24 h before and immediately after cerclage, and every 2 weeks thereafter, until 32 weeks. Patients are instructed to empty their bladder beforehand. The probe is gently introduced until resistance is felt, then slightly withdrawn to avoid cervical compression. When funneling is observed, cervical length is measured from the innermost point of the funnel to the external cervical os. Three separate measurements are recorded during each scan. The mean value of these measurements was used for the current analysis.

Data on obstetrical and perinatal outcomes were also collected, including the incidence of spontaneous preterm delivery, gestational age at delivery, birthweight and cesarean delivery rate, umbilical artery pH, Apgar scores, and neonatal intensive care unit admission.

### Statistics

For statistical analysis, quantitative variables were presented as means and standard deviations, or as medians and interquartile ranges, depending on the data distribution. Qualitative variables were reported as frequencies and percentages. Continuous variables were compared using the *t *test, while categorical variables were analyzed using the Chi-square test or Fisher’s exact test, as appropriate. Rank sum analysis (Mann–Whitney test) was applied when necessary. A *p* value of less than 0.05 was considered statistically significant.

Receiver operating characteristic (ROC) curve analysis was used to assess the predictive performance of cervical length measurements at different gestational age intervals for PTB before 32 weeks. For each time interval, the area under the curve (AUC) with 95% confidence intervals (CI) and *p *values were reported. Cutoff values were determined separately for optimal sensitivity and optimal specificity.

### Ethics

The protocol of the study was approved by the local Institutional Review Board (Helsinki Committee) of our medical center.

## Results

During the study period, 157 women were eligible for inclusion, of whom 109 underwent prophylactic cerclage and 46 received emergency cerclage.

### Prophylactic cerclage group (*n* = 109)

Among the women who underwent prophylactic cerclage, 23 experienced spontaneous PTB < 37 weeks and 86 delivered at term. Statistically significant differences were not found between these groups, in maternal age, parity, ethnicity, history of PTB, or progesterone treatment. Among the women with spontaneous PTB compared with term births, the mean gestational age at delivery was lower (32.8 vs. 38.7 weeks, *p* < 0.001), the mean birthweight was lower (2068.8 vs. 3212.8 g, *p* < 0.001), and the proportion of neonatal intensive care unit admissions was higher (65.2% vs. 4.7%, *p* < 0.001) (Table 1).

**Table 1:** Baseline characteristics of women who underwent prophylactic cerclage

	Spontaneous preterm birth (*N* = 23)	Term birth (*N* = 86)	*p* value
Maternal age, mean (± SD)	30.0 (± 6.0)	30.8 (± 5.2)	0.517
Gravity, median (IQR)	4 (2–6)	4 (3–5)	0.686
Parity, median (IQR)	1 (2–4)	2 (2–3)	0.268
Ethnicity, *n*. (%)			0.811
Arabs	14 (60.9)	55 (64.0)	
Jews	9 (39.1)	31 (36.0)	
GBS carrier, *n*. (%)	3 (13.0)	13 (15.1)	1.00
Smoking, *n*. (%)	1 (4.3)	6 (7.0)	1.00
Pre-gestational DM, *n*. (%)	0 (0)	1 (1.2)	1.00
Gestational DM, *n*. (%)	5 (21.7)	14 (16.3)	0.544
Chronic HTN, *n*. (%)	0 (0)	1 (1.2)	1.00
History of PTB, *n*. (%)	13 (56.5)	52 (60.5)	0.812
Previous conization, *n*. (%)	8 (9.3)	3 (13.0)	0.697
Second trimester pregnancy loss, *n*. (%)	2 (8.7)	31 (36.0)	0.011
Progesterone treatment, *n*. (%)	10 (43.5)	42 (48.8)	0.815
GA at cerclage, mean (± SD)	14.0 (± 1.6)	14.1 (± 1.1)	0.885
GA at delivery, mean (± SD)	32.8 (± 3.7)	38.7 (± 1.0)	< 0.001
Birthweight, mean (± SD)	2068.8 (± 744.6)	3212.8 (445.7 ±)	< 0.001
Spontaneous PTB < 32w, *n*. (%)	9 (39.1)	0 (0)	< 0.001
Cesarean delivery, *n*. (%)	3 (13.0)	30 (34.9)	0.071
5-min Apgar score < 7, *n*. (%)	0 (0)	0 (0)	NA
Umbilical artery pH < 7.15, *n*. (%)	0 (0)	1 (3.6)	1.00
NICU admission, *n*. (%)	15 (65.2)	4 (4.7)	< 0.001

*SD* standard deviation, *IQR* interquartile range, *GBS* group B streptococcus, *BMI* body mass index, *DM* diabetes mellitus, *HTN* hypertension, *PTB* preterm birth, *BMI* body mass index, *CL* cervical length, *GA* gestational age, *NICU* neonatal intensive care unit

**Fig. 1:**
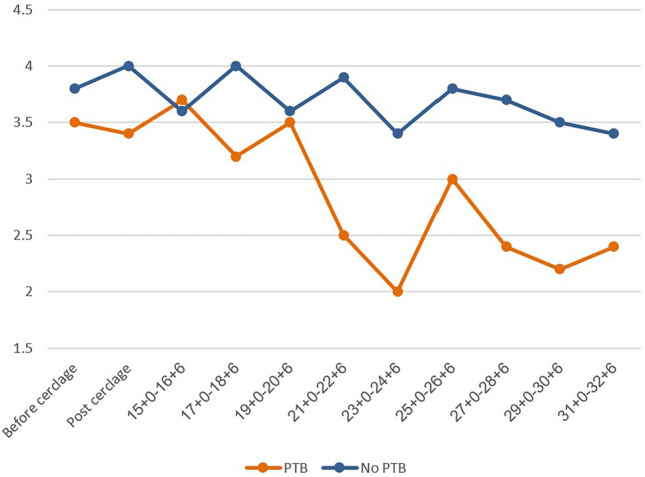
Serial cervical length in women with vs without preterm birth < 37 weeks after prophylactic cerclage, *PTB* preterm birth

Cervical length measurements before and immediately after cerclage were similar between women who delivered preterm and at term. However, the median cervical length was significantly shorter in the preterm group at several points following cerclage placement. Notably, differences were observed at 21 + 0–22 + 6 weeks (2.5 vs. 3.9 cm, *p* = 0.042), 23 + 0–24 + 6 weeks (2.0 vs. 3.4 cm, *p* = 0.016), and 25 + 0–26 + 6 weeks (3.0 vs. 3.8 cm, *p* = 0.042), and continued through 31 + 0–32 + 6 weeks (2.4 vs. 3.4 cm, *p* = 0.015). Funneling was also more common in the preterm group (39.1% vs. 12.8%, *p* = 0.012). These findings are summarized in Table 2 and illustrated in Fig. 1.

**Table 2:** Serial cervical length measurements following prophylactic cerclage in women with and without subsequent spontaneous preterm birth < 37 weeks

	Spontaneous preterm birth (*N* = 23)	Term birth (*N* = 86)	*p* value
Funneling, *n*. (%)	9 (39.1)	11 (12.8)	0.012
CL before cerclage	3.5 (2.8–4.0)	3.8 (3.3–4.4)	0.095
CL post-cerclage	3.4 (2.7–4.3)	4.0 (3.2–4.2)	0.341
CL 15 + 0–16 + 6w	3.7 (3.7–3.8)	3.6 (3.1–4.0)	0.712
CL 17 + 0–18 + 6w	3.2 (2.5–4.3)	4.0 (3.4–4.2)	0.486
CL 19 + 0–20 + 6w	3.5 (2.8–3.9)	3.6 (3.2–3.9)	0.517
CL 21 + 0–22 + 6w	2.5 (1.6–3.7)	3.9 (3.1–4.1)	0.042
CL 23 + 0–24 + 6w	2.0 (1.5–2.9)	3.4 (2.9–4.0)	0.016
CL 25 + 0–26 + 6w	3.0 (1.9–3.6)	3.8 (2.7–3.9)	0.042
CL 27 + 0–28 + 6w	2.4 (1.8–3.5)	3.7 (2.9–4.2)	0.019
CL 29 + 0–30 + 6w	2.2 (1.5–3.4)	3.5 (2.7–4.0)	0.015
CL 31 + 0–32 + 6w	2.4 (2.0–2.7)	3.4 (3.0–4.0)	0.015

*CL* cervical length

*Cervical length values are presented as median (interquartile range)

**Fig. 2:**
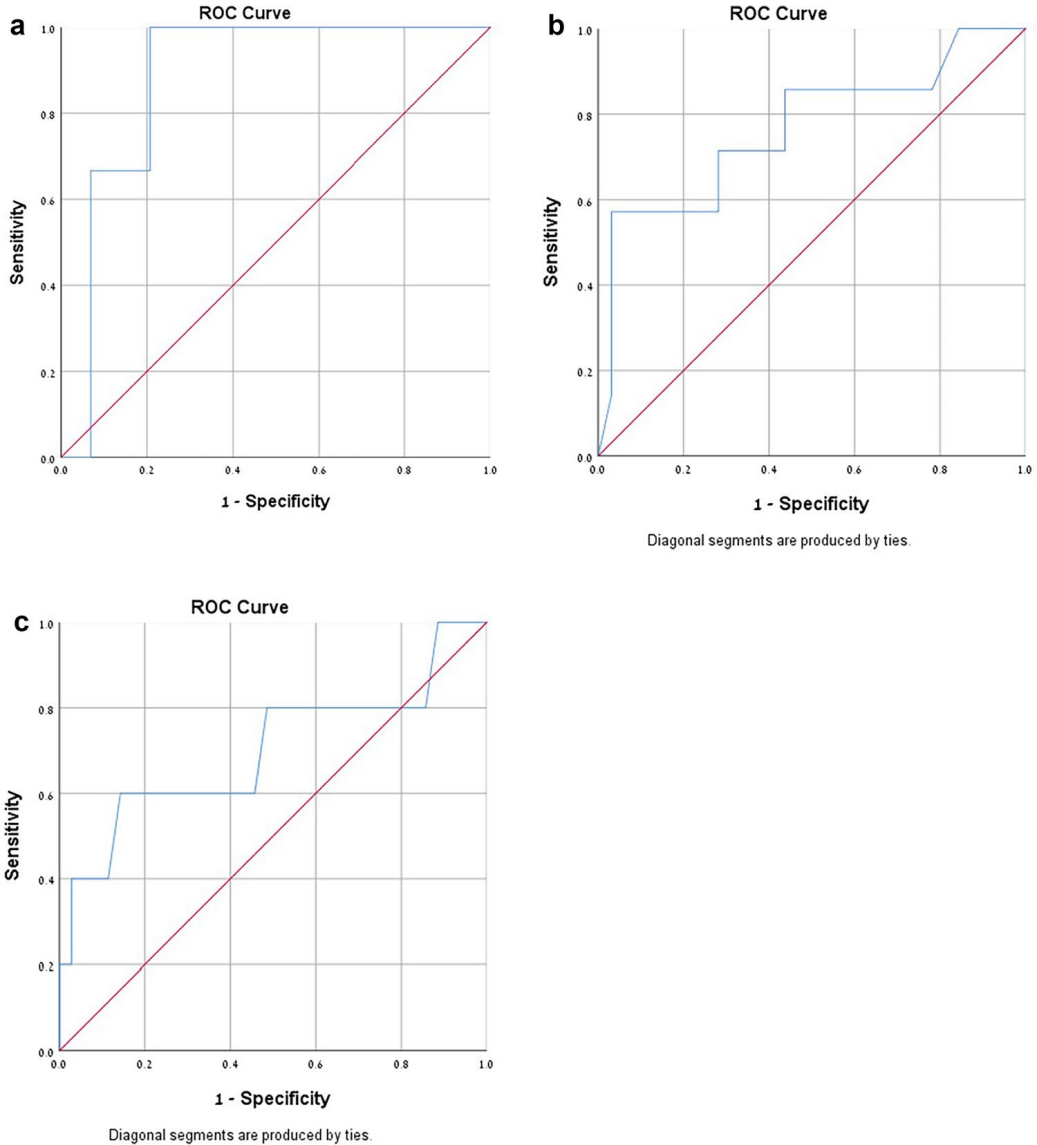
ROC curves for cervical length in predicting preterm birth before 32 weeks following prophylactic cerclage. **A** 23 + 0–24 + 6 weeks, **B** 25 + 0–26 + 6 weeks, **C** 27 + 0–28 + 6 weeks

ROC analysis showed that cervical length at 23 + 0 to 24 + 6 weeks yielded an AUC of 0.97 (95% CI 0.89–1.000, *p* = 0.011). The cutoff of ≤ 2.8 cm provides 100% sensitivity and 94.7% specificity. A stricter cutoff of ≤ 2.1 cm increases specificity to 100%, with 33.3% sensitivity. Cutoff values and performance measures for the 25 + 0 to 26 + 6, and 27 + 0 to 28 + 6 week intervals are presented in Table 3. Figure 2 presents the ROC curves for cervical length measurements at different gestational age windows in predicting preterm birth before 32 weeks.

**Table 3:** Receiver operating characteristics curve analysis of cervical length for predicting preterm birth < 32 weeks after prophylactic cerclage

Gestational age interval	Area under the curve (95% CI)	*p* value	Best sensitivity cutoff	Best specificity cutoff
23 + 0 to 24 + 6	0.97 (0.89–1.000)	0.011	≤ 2.8 cm Sensitivity 100% Specificity 94.7%	≤ 2.1 cm Specificity 100% Sensitivity 33.3%
25 + 0 to 26 + 6	0.89 (0.76–1.00)	0.030	≤ 2.7 cm Sensitivity 100% Specificity 79.3%	≤ 1.5 cm Specificity 93.1% Sensitivity 33.3%
27 + 0 to 28 + 6	0.77 (0.55–0.99)	0.029	≤ 4.1 cm Sensitivity 100% Specificity 81.3%	≤ 1.6 cm Specificity 96.9% Sensitivity 14.3%

*CI* confidence interval

In multivariable logistic regression, shorter cervical length at 23 + 0 to 24 + 6 weeks was significantly associated with spontaneous PTB < 37 weeks (adjusted odds ratio [aOR] 4.13, 95% CI 1.23–13.89, *p* = 0.021). A similar association was observed at 25 + 0 to 26 + 6 weeks (aOR 3.39, 95% CI 1.08–10.64, *p* = 0.037). At this later time point, cervical length also independently predicted PTB < 32 weeks (aOR 4.76, 95% CI 1.20–19.60, *p* = 0.027). Other variables, including a history of PTB, progesterone use, and gestational age at cerclage, were not significantly associated with the outcome.

### Emergency cerclage group (*n* = 46)

Of the 46 women in the emergency cerclage group, 19 (41.3%) underwent cerclage placement at ≥ 20 weeks of gestation. Of the women who underwent emergency cerclage, 10 delivered preterm and 36 reached term. PTB birth was associated with lower neonatal birthweight (2220.0 vs. 3192.8 g, *p* < 0.001) and a markedly higher rate of neonatal intensive care unit admissions (70.0% vs. 11.1%, *p* = 0.001). Other baseline characteristics, including maternal age, parity, prior PTB, group B streptococcus carriage, and progesterone use, were comparable between the groups (Table 4).

**Table 4:** Baseline characteristics of women who underwent emergency cerclage

	Spontaneous preterm birth (*N* = 10)	Term birth (*N* = 36)	*p* value
Maternal age, mean (± SD)	33 (± 5.3)	31.1 (5.4)	0.168
Gravity, median (IQR)	3 (2–6)	3 (2–5)	0.432
Parity, median (IQR)	1 (1–3)	2 (1–3)	0.5392
Ethnicity, *n*. (%)			0.475
Arabs	4 (40.0)	15 (41.7)	
Jews	6 (60.)	21 (58.3)	
GBS carrier, *n*. (%)	2 (20)	2 (5.6)	0.201
Smoking, *n*. (%)	1 (10.0)	3 (8.3)	1.00
Pre-gestational DM, *n*. (%)	0 (0)	3 (8.3)	0.585
Gestational DM, *n*. (%)	5 (50.0)	6 (16.7)	0.049
Chronic HTN, *n*. (%)	0 (0)	0 (0)	NA
History of PTB, *n*. (%)	6 (60.0)	8 (22.2)	0.047
Previous conization, *n*. (%)	0 (0)	4 (11.1)	0.562
Progesterone treatment, *n*. (%)	7 (70.0)	15 (41.7)	0.159
GA at cerclage, mean (± SD)	19.2 (± 2.9)	18.1 (± 3.4)	0.492
GA at delivery, mean (± SD)	33.2 (± 3.9)	38.6 (± 2.1)	< 0.001
Birthweight, mean (± SD)	2220.0 (± 812.6)	3192.8 (± 426.7)	< 0.001
Spontaneous PTB < 32w, *n*. (%)	3 (30.0)	0 (0)	0.008
Cesarean delivery, *n*. (%)	4 (40.0)	13 (36.1)	1.00
5-min Apgar score < 7, *n*. (%)	1 (10)	1 (2.8)	0.391
Umbilical artery pH < 7.15, *n*. (%)	0 (0)	0 (0)	NA
NICU admission, *n*. (%)	7 (70.0)	4 (11.1)	0.001

*SD* standard deviation, *IQR* interquartile range, *GBS* group B streptococcus, *BMI* body mass index, *DM* diabetes mellitus, *HTN* hypertension, *PTB* preterm birth, *BMI* body mass index, *CL* cervical length, *GA* gestational age, *NICU* neonatal intensive care unit

Cervical length measurements after cerclage, and also at subsequent time points up to 32 + 6 weeks, were also comparable between the groups (Table 5). However, the median cervical lengths at 23 + 0–24 + 6 and 25 + 0–26 + 0 weeks were significantly shorter among women with spontaneous PTB before 32 weeks than among those who delivered at term. For the preterm compared to the term group, the median cervical length was 1.4 cm versus 3.2 cm, *p* = 0.049 at 23 + 0–24 + 6 weeks; and 1.5 cm versus 2.3 cm, one-sided *p* value = 0.041 at 25 + 0–26 + 0 weeks.

**Table 5:** Serial cervical length measurements following emergency cerclage in women with and without subsequent spontaneous preterm birth < 37 weeks

	Spontaneous preterm birth (*N* = 10)	Term birth (*N* = 36)	*p* value
Funneling, *n*. (%)	6 (60.0)	25 (69.4)	0.706
CL before cerclage	1.2 (0.4–1.8)	1.7 (1.2–2.4)	0.036
CL post-cerclage	3.0 (2.4–3.2)	3.2 (2.4–3.5)	0.964
CL 19 + 0–20 + 6w	1.5 (1.5–1.5)	2.3 (2.1–2.3)	0.500
CL 21 + 0–22 + 6w	1.8 (1.5–1.8)	1.6 (0.3–2.5)	0.933
CL 23 + 0–24 + 6w	2.8 (1.3–3.3)	3.2 (2.1–3.3)	0.381
CL 25 + 0–26 + 6w	1.9 (1.2–3.4)	2.3 (1.7–3.2)	0.536
CL 27 + 0–28 + 6w	1.9 (1.3–2.9)	2.5 (2.1–3.5)	0.264
CL 29 + 0–30 + 6w	2.6 (1.3–2.6)	2.2 (1.7–2.5)	0.517
CL 31 + 0–32 + 6w	2.7 (1.6–3.2)	2.4 (1.7–2.9)	0.615

*CL* cervical length

*Cervical length values are presented as median (interquartile range)

**Fig. 3:**
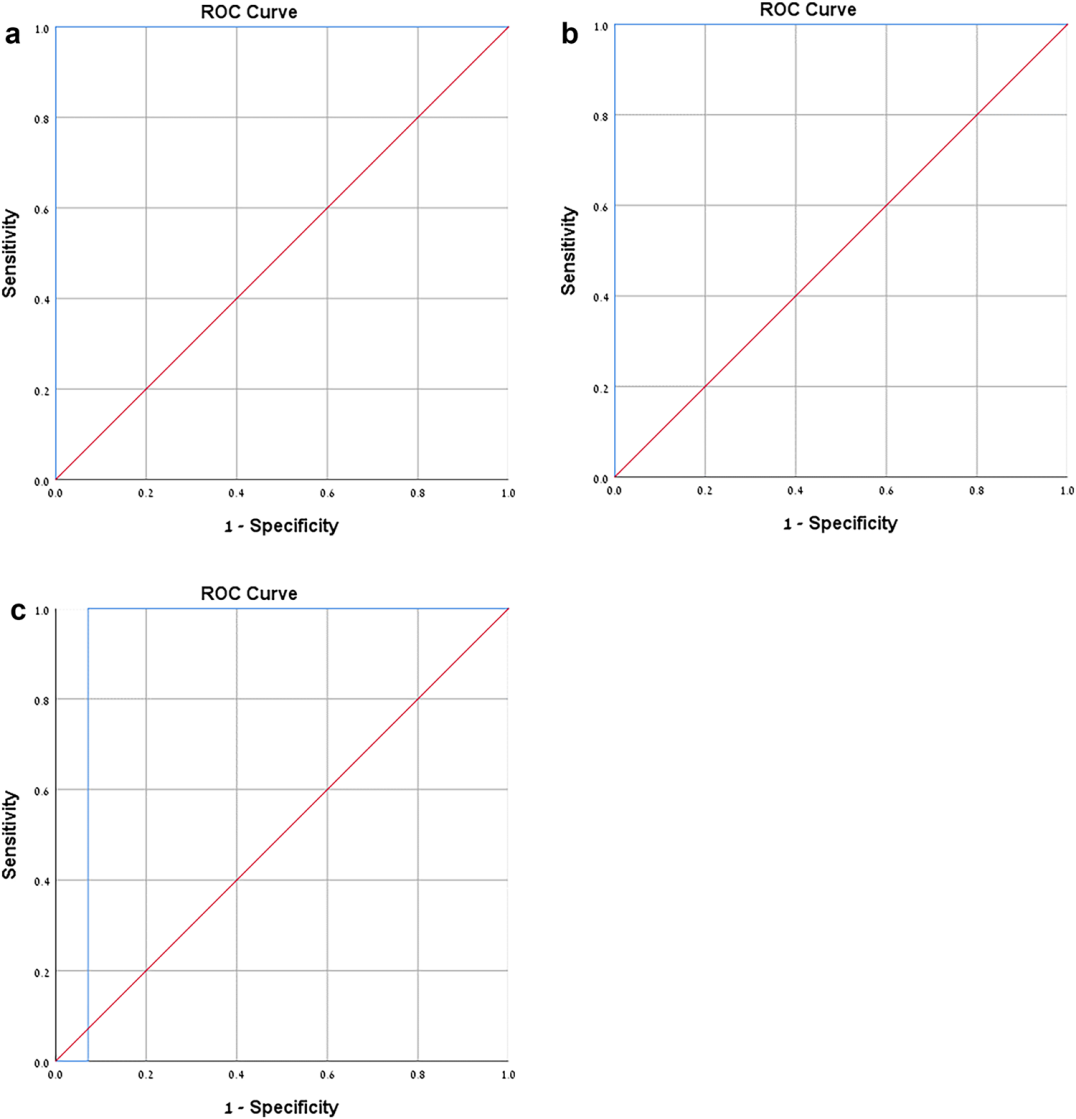
ROC curves for cervical length in predicting preterm birth before 32 weeks following emergency cerclage. **A** 23 + 0–24 + 6 weeks, **B** 25 + 0–26 + 6 weeks, **C** 27 + 0–28 + 6 weeks

ROC analysis showed that a cervical length of ≤ 1.95 cm at 23 + 0 to 24 + 6 weeks predicted PTB < 32 weeks, with both 100% sensitivity and specificity (AUC 1.00, *p* = 0.040). Additional cutoffs for maximal specificity or reduced sensitivity at this and later time points (25 + 0 to 26 + 6 weeks and 27 + 0 to 28 + 6 weeks) are detailed in Table 6. Figure 1 presents the ROC curves for cervical length measurements at different gestational age windows in predicting preterm birth before 32 weeks (Fig. 3).

**Table 6:** Receiver operating characteristics curve analysis of cervical length for predicting preterm birth < 32 weeks after emergency cerclage

Gestational age interval	Area under the curve (95% CI)	*p* value	Best sensitivity cutoff	Best specificity cutoff
23 + 0 to 24 + 6	1.00 (1.00–1.00)	0.040	≤ 1.95 cm Sensitivity 100% Specificity 100%	≤ 1.43 cm Sensitivity 50% Specificity 100%
25 + 0 to 26 + 6	0.83 (0.61–1.00)	0.0415*	≤ 2.10 cm Sensitivity 100% Specificity 63.2%	≤ 1.25 cm Specificity 100% Sensitivity 33%
27 + 0 to 28 + 6	0.93 (0.79–1.00)	0.165	≤ 1.35 cm Sensitivity 100% Specificity 92.9%	≤ 0.10 cm Sensitivity 100% Specificity 0%

*CI* confidence interval

*p value one-sided

Among women who experienced spontaneous PTB birth before 32 or 37 weeks, cervical length measurements at various time points did not significantly differ between those who received prophylactic versus emergency cerclage.

Preterm birth < 32 weeks occurred in 9 of 109 (8.3%) women with prophylactic cerclage and 3 of 46 (6.5%) women with emergency cerclage (*p* = 1.00). Preterm birth < 37 weeks occurred in 23 of 109 (21.1%) and 10 of 46 (21.7%) women, respectively (*p* = 1.00).

## Discussion

The findings of this study demonstrate that cervical length assessed during serial follow-up after cerclage placement is a significant predictor of spontaneous PTB. Both among women who underwent prophylactic and emergency cerclage, those who ultimately delivered preterm exhibited more pronounced cervical shortening during follow-up. Cervical length assessment has long served as a cornerstone in the prediction of spontaneous PTB. In women without cerclage, a short cervix—particularly below 25 mm before 24 weeks—is a well-established marker of increased PTB risk [9]. However, the utility of cervical length measurement after cerclage placement has been less clearly defined. Theoretical concerns include potential distortion of cervical anatomy, artificial lengthening due to the stitch, or altered mechanical properties of the cervix following intervention. These factors could potentially confound the interpretation of sonographic measurements post-cerclage [10]. Our findings align with studies that highlighted the prognostic value of cervical length following cerclage. Dijkstra et al. showed that among women with history-indicated cerclage, cervical length was significantly longer among those who delivered at term [10]. Meaningful differences were already evident from 20–23 weeks and persisted throughout gestation. In contrast, among women with urgent cerclage, cervical length only became predictive of PTB at 28–32 weeks. This suggests that early mid-trimester surveillance may be less informative in this population. However, among our women with emergency cerclage, cervical length measured at both 23–24 + 6 and 25–26 weeks was already significantly shorter among those who later delivered before 32 weeks. This discrepancy may reflect different outcome definitions, as Dijkstra et al. focused on birth before 37 weeks [10], whereas we examined a more severe outcome of PTB before 32 weeks. Pils et al. also found that cervical length was not predictive of outcome in women with emergency cerclage, but their primary endpoint was delivery before 35 weeks [11]. This further highlights that the predictive utility of cervical length may depend on the severity of the outcome assessed.

The role of TVS cervical length assessment shortly after cerclage placement remains debated. In our cohort, postoperative cervical length did not differ significantly between women who ultimately delivered preterm and those who delivered later, whether the cerclage was urgent or prophylactic. However, shorter CL following ultrasound-indicated cerclage was previously shown to be associated with higher risks of PTB and adverse neonatal outcomes [12–14]. These differences may reflect variations in cerclage indication, timing of measurement, or the chosen gestational age thresholds for defining PTB.

Other studies have explored the predictive value of post-cerclage ultrasound findings. Battarbee et al. conducted a retrospective study that included McDonald, Shirodkar, and abdominal cerclages, placed for both prophylactic and emergent indications [15]. Cervical length measured between 16 + 0 and 25 + 6 weeks identified several features as significantly associated with PTB before 34 and 37 weeks. The features included short cervical length below the stitch, inner-third stitch location, funneling past the stitch, sludge, and a straight cervical canal. Cochrane et al. reported that TVS cervical length measured within 2 weeks of cerclage placement of < 2.0 cm was significantly associated with spontaneous PTB (adjusted OR 6.3, 95% CI 2.2–18.8, *p* < 0.001) [16]. However, *a* < 2.5 cm threshold was not statistically significant. The findings of Ridout et al. suggested that combining TVS cervical length measurement with quantitative fetal fibronectin testing may enhance the prediction of spontaneous PTB before 30 weeks in high-risk women with cervical cerclage [5]. An interesting observation was that cervical shortening among those who experienced PTB became more pronounced after 20 weeks of gestation. Findings are consistent with those reported by Dijkstra et al. [10] The weight of the growing pregnancy, particularly after mid-gestation, may contribute to progressive funneling and effacement despite mechanical support.

In our multivariable analysis, cervical length during follow-up emerged as a significant predictor of spontaneous PTB, whereas progesterone treatment was not independently associated with reduced risk. This finding contrasts with evidence suggesting a benefit for vaginal progesterone, particularly when used alongside cerclage in high-risk women [17]. Differences in treatment timing, adherence, and selection criteria for progesterone use may explain the discrepancy between the results.

We found that preterm birth rates before 32 and 37 weeks were similar between women who received prophylactic cerclage and those who underwent emergency placement, thus collaborating prior studies. Guzman et al. observed comparable outcomes following prophylactic and ultrasound-indicated cerclage performed after cervical shortening [13]. Nelson et al. reported similar delivery timing in women treated for sonographically detected shortening versus those managed prophylactically [18]. Our findings may be explained by our individualized management approach. Women who presented with cervical shortening or mild dilation were admitted for 24–48 h of observation to exclude early infection or inevitable pregnancy loss before cerclage placement, thus ensuring appropriate candidate selection. Our results suggest that a selective, observation-based approach can yield outcomes equivalent to prophylactic cerclage, thus supporting the safety of delaying intervention until cervical changes are documented.

This study has several strengths. The setting of a single tertiary center ensures uniform management protocols for PTB prevention and minimizes treatment-related variability. The cohort included only women who received a McDonald cerclage, avoiding heterogeneity related to different cerclage techniques. Additionally, the cohort was well-characterized, and included women who underwent prophylactic and emergency cerclage, thus enabling meaningful subgroup analysis. The use of standardized TVS measurements at regular intervals enhanced the consistency and reliability of the findings. Furthermore, the detailed ROC analyses offer clinically useful cutoff values that may support risk stratification in practice. The analysis of performance for optimal sensitivity and specificity supports individualized decision-making depending on clinical priorities.

Several limitations must be acknowledged. The retrospective design introduces potential biases related to selection and data completeness. While cervical length measurements were conducted according to departmental protocol, some interobserver variability may have occurred, which could have affected measurement consistency. Second, although all emergency cerclages in this study were ultrasound-indicated and had a measurable cervical length, this group remained clinically heterogeneous, including women with progressive cervical shortening during surveillance or incidentally detected short cervix at routine ultrasound. This heterogeneity may have influenced outcome estimates and should be considered when interpreting the results. The small number of PTBs limits the statistical power of some analyses and may overestimate the performance of identified cutoffs. Additionally, this study did not include inflammatory biomarkers or cervical elastography, which might further improve risk stratification in future research. Future studies should aim to validate these aspects, and to examine whether combining cervical length with other clinical or biochemical markers could further enhance predictive accuracy. Another research question is whether targeted interventions based on short cervical length after cerclage improve maternal or neonatal outcomes.

In conclusion, cervical length measured after cerclage placement remains a valuable predictor of spontaneous PTB. These measurements may help tailor surveillance strategies and inform timely interventions in women at risk of PTB. Incorporating routine cervical length assessment during the second trimester, even after cerclage, may help optimize outcomes for these women.

## Data Availability

The data that support the findings of this study are not publicly available due to their containing information that could compromise the privacy of the research participants but are available from the corresponding author [IS] upon reasonable request. Data may be obtained from the corresponding author upon reasonable request.
